# Transmission of Artemisinin-Resistant Malaria Parasites to Mosquitoes under Antimalarial Drug Pressure

**DOI:** 10.1128/AAC.00898-20

**Published:** 2020-12-16

**Authors:** Kathrin Witmer, Farah A. Dahalan, Michael J. Delves, Sabrina Yahiya, Oliver J. Watson, Ursula Straschil, Darunee Chiwcharoen, Boodtee Sornboon, Sasithon Pukrittayakamee, Richard D. Pearson, Virginia M. Howick, Mara K. N. Lawniczak, Nicholas J. White, Arjen M. Dondorp, Lucy C. Okell, Kesinee Chotivanich, Andrea Ruecker, Jake Baum

**Affiliations:** a Department of Life Sciences, Imperial College London, London, United Kingdom; b London School of Hygiene and Tropical Medicine, London, United Kingdom; c Medical Research Council Centre for Global Infectious Disease Analysis, Department of Infectious Disease Epidemiology, Imperial College London, London, United Kingdom; d Mahidol Oxford Tropical Medicine Research Unit, Faculty of Tropical Medicine, Mahidol University, Bangkok, Thailand; e Department of Clinical Tropical Medicine, Faculty of Tropical Medicine, Mahidol University, Bangkok, Thailand; f Wellcome Sanger Institute, Hinxton, United Kingdom; g Centre for Tropical Medicine and Global Health, Nuffield Department of Medicine, University of Oxford, Oxford, United Kingdom

**Keywords:** *Anopheles stephensi*, *Kelch13*, *Plasmodium falciparum*, artemisinin combination therapies (ACTs), gametocytes, multidrug-resistant malaria, transmission blocking

## Abstract

Resistance to artemisinin-based combination therapy (ACT) in the Plasmodium falciparum parasite is threatening to reverse recent gains in reducing global deaths from malaria. While resistance manifests as delayed parasite clearance in patients, the phenotype can only spread geographically via the sexual stages and mosquito transmission. In addition to their asexual killing properties, artemisinin and its derivatives sterilize sexual male gametocytes. Whether resistant parasites overcome this sterilizing effect has not, however, been fully tested. Here, we analyzed P. falciparum clinical isolates from the Greater Mekong Subregion, each demonstrating delayed clinical clearance and known resistance-associated polymorphisms in the *Kelch13* (PfK13^var^) gene. As well as demonstrating reduced asexual sensitivity to drug, certain PfK13^var^ isolates demonstrated a marked reduction in sensitivity to artemisinin in an *in vitro* male gamete formation assay. Importantly, this same reduction in sensitivity was observed when the most resistant isolate was tested directly in mosquito feeds. These results indicate that, under artemisinin drug pressure, while sensitive parasites are blocked, resistant parasites continue transmission. This selective advantage for resistance transmission could favor acquisition of additional host-specificity or polymorphisms affecting partner drug sensitivity in mixed infections. Favored resistance transmission under ACT coverage could have profound implications for the spread of multidrug-resistant malaria beyond Southeast Asia.

## Abstract (precis)

Resistance to artemisinin-based combination therapy (ACT) in the Plasmodium falciparum parasite is threatening to reverse recent gains in reducing global deaths from malaria. While resistance manifests as delayed parasite clearance in patients, the phenotype can only spread geographically via the sexual stages and mosquito transmission. In addition to their asexual killing properties, artemisinin and its derivatives sterilize sexual male gametocytes. Whether resistant parasites overcome this sterilizing effect has not, however, been fully tested.

## INTRODUCTION

Malaria kills more than 400,000 people each year (1). While there has been a marked reduction in global rates of malaria disease since the new millennium, progress has stalled and recently even reversed in some regions (1). A critical factor threatening future gains in malaria control is the emergence and spread of drug resistance in the most virulent parasite Plasmodium falciparum (2). Of most concern is the reported spread of resistance to frontline artemisinin-based drugs in the Greater Mekong Subregion (GMS) of Southeast Asia (SEA) (3–5). Artemisinin has revolutionised treatment for severe malaria. The drug acts rapidly to clear the clinical symptoms of malaria by killing the asexual parasite in host red blood cells (RBCs). Although a precise mechanism of action is contested, it is thought that intracellular iron-mediated activation of artemisinin arising from parasite metabolism of hemoglobin causes the drug to be both highly reactive and consumed rapidly in the process of its action (6). Consequently, use of artemisinin or its derivatives requires coformulation with longer-lasting partner drugs as artemisinin-based combination therapies (ACTs). In recent years, however, resistance to both artemisinin and partner drugs, including piperaquine and mefloquine, has increased in prevalence throughout SEA (4, 7–10). The spread of such multidrug resistant parasites beyond the GMS could prove catastrophic for global malaria control (11, 12).

Resistance to artemisinin is strongly associated with nonsynonymous single nucleotide polymorphisms (SNPs) in the propeller domain of P. falciparum Kelch13 (PfK13) (13) a protein with a role in hemoglobin endocytosis from the host cell (14, 15). Based on SNP analysis, several PfK13 variants (PfK13^var^) have been defined displaying different degrees of delayed parasite clearance in patients under ACT treatment. PfK13^var^ include mutually exclusive SNPs giving rise to amino acid changes C580Y, R539T, I543T and Y493H (4, 8, 16, 17). While the precise mechanism by which different PfK13^var^ determine resistance remains ill-defined (6), PfK13^var^ parasites show an upregulation in the unfolded protein response (18). In asexual ring stages, the presence of PfK13^C580Y^ results in the reduced endocytotic uptake of hemoglobin, potentially leading to reduced intracellular activity of artemisinin (14, 15). Persistence of parasites in the blood of infected individuals will lead to their delayed clearance and ultimately treatment failure. Among PfK13 polymorphisms, the PfK13^C580Y^ genotype is the most widely spread variant currently circulating in SEA (7, 8).

Drug resistance and its spread are traditionally seen through the prism of disease, which, in the case of malaria, is the asexual replicative stages of the life cycle carried in blood circulation. However, resistance can only spread with passage of the parasite through the mosquito, a fundamental step in the *Plasmodium* life cycle (19). Transmission of malaria parasites is solely mediated by nonpathogenic sexual stages called gametocytes. These gametocytes mature over the course of 10 to 12 days and are the only stages infectious to mosquitoes (19). During a mosquito blood feed, male and female gametocytes are taken up and activated in the mosquito midgut into male and female gametes. These activated gametes then fertilize and form a motile zygote (ookinete) that infects the midgut epithelium, forming an oocyst on the gut lining (20). The oocyst eventually bursts releasing sporozoites that can be transmitted back into humans during a subsequent bite from an infected mosquito.

While the activity of artemisinin derivatives on asexual stage parasites is well known, one overlooked property of these drugs is their ability to target sexual stages, specifically their ability to block the activation of male gametes (exflagellation), which underpins transmission (21, 22). This raises the question as to whether artemisinin-resistant parasites are also resistant to this sterilizing effect in the context of transmission to the mosquito. Here, we sought to test how clinical isolates with demonstrated tolerance or treatment delay against artemisinin (i.e., asexual stage growth) fair in their transmissibility through the mosquito under artemisinin coverage. We showed that male gametocytes of a PfK13^C580Y^ isolate are activated under drug pressure and are thus able to infect mosquitoes under artemisinin treatment compared to a sensitive control. These findings have important implications for modeling the spread of resistance across geographical regions. This additional effect of artemisinin resistance on transmission emphasizes the need for future combination therapies that include a transmission-blocking component if we are to stem the spread of resistance beyond the GMS.

## RESULTS

### Selection and adaptation of Southeast Asian P. falciparum clinical isolates for *in vitro* study.

P. falciparum clinical isolates that successfully adapted to long-term culture (Chotivanich, unpublished data) were derived from a previous, multicenter, open-label, randomized trial collecting samples from patients with acute, uncomplicated malaria (16). All isolates were sequenced and assessed for multiplicity of infection (MOI) at point-of-care and determined to be sufficiently homozygous to make cloning (and potentially loss of ability to form gametocytes) unnecessary (23). Among isolates, five were followed further based on their ability to form functional mature gametocytes *in vitro*. These were compared to the standard laboratory control parasite strain NF54. Each was validated by PCR, confirming the five clinical isolates have variant polymorphisms in PfK13 (Table 1). Each P. falciparum isolate was tested *ex vivo* for sensitivity to the artemisinin derivative artesunate using the 24-h trophozoite maturation inhibition assay (TMI). The TMI assesses the effect of artemisinin derivatives on the maturation of asexual ring stages to trophozoites in comparison to an artemisinin-sensitive control (24). While PfK13^var^ isolates presented a wide range of TMI 50% inhibitory concentration (IC_50_) values, they all showed increased resistance to artesunate compared to a PfK13^WT^ culture-adapted Thai and non-gametocyte-producing laboratory strain, TM267 (Table 1).

**TABLE 1:** Characteristics of the P. falciparum field isolates presented in this study

Clinical isolate code	Year	Origin	Parasite clearance half-life (hours)^*a*^	Mean (SD) artesunate TMI IC_50_ (nM)^*b*^	PfK13 genotype^*c*^
TM267	1995	Thailand	ND	1.82 (1.3–2.3)^*b*^	WT (24)
ARN1G	May 2011–April 2013	Ranong, Thailand	7.1	17.69 (0.26)	G449A
APS2G	May 2011–April 2013	Srisaket, Thailand	6.1	24.19 (4.16)	R539T
APS3G	May 2011–April 2013	Srisaket, Thailand	6.2	33.56 (1.04)	R539T
APL4G	May 2011–April 2013	Pailin, West Cambodia	5.1	17.17 (2.6)	C580Y
APL5G	May 2011–April 2013	Pailin, West Cambodia	7.7	13.53 (6.24)	C580Y

a ND, not done.

b IC_50_ values of field isolates assayed with three independent biological replicates using the trophozoite maturation inhibition assay (TMI) (24). Standard deviations are indicated in brackets (SD). The P. falciparum TM267 assay control isolate originates from a previous study (24) with others from (16). For the wild-type strain TM257, the parentheses contain the 95% confidence interval.

c As determined by PCR.

In addition to the PfK13 genotype, the genetic background of each parasite isolate was investigated to explore whether additional mutations might be present, such as those associated with other drug resistance phenotypes. Whole-genome sequencing analysis was completed for each, confirming different PfK13 genotypes (Table 2). In addition, multiple previously reported mutations in genes associated with various drug sensitivities were found among PfK13^var^ isolates (as reviewed in reference 2) (Table 2). Mutations were found in the chloroquine resistance transporter (*pfcrt*), agreeing with reported mutations found in some parasites following ACT treatment (25). None of the isolates, however, carried mutations in *pfcrt* associated with increased DHA-piperaquine treatment failure (7, 8, 26) or piperaquine resistance *in vitro* (27, 28). In addition, all isolates carried mutations in four genes that are associated with the PfK13^C580Y^ haplotype, namely, *fd* (ferredoxin), *arps10* (apicoplast ribosomal protein S10), *mdr2* (multidrug resistance protein 2), and *crt* (chloroquine resistance transporter) (Table 2) (17).

**TABLE 2:** Summary molecular markers associated with antimalarial drug resistance for isolates used in this study

Gene ID	Product	Amino acid change^*a*^^,^^*b*^	Isolate^*c*^				
			ARN1G	APS2G	APS3G	APL5G	APL4G
PF3D7_1343700	Kelch protein K13	G449A	x	−	−	−	−
		R539T	−	x	x	−	−
		C580Y	−	−	−	x	x
PF3D7_0112200	Multidrug resistance-associated protein 1	H191Y	x	X	x	x	x
		K202E	−	−	−	x	−
		N325S	−	−	−	−	−
		S437A	x	X	x	x	x
		I876V	x	−	−	x	x
		F1390I	x	−	−	x	x
PF3D7_0417200	Bifunctional dihydrofolate reductase-thymidylate synthase	N51I	x	x	x	x	x
		C59R	x	x	x	x	x
		S108N	x	x	x	x	x
		I164L	−	−	−	−	x
PF3D7_0523000	Multidrug resistance protein 1	Y184F	−	x	x	x	x
		*amplification*	−	−	−	x	−
PF3D7_0709000	Chloroquine resistance transporter	K76T	x	x	x	x	x
		*T93S*^*a*^	−	−	−	−	−
		H97Y	−	−	−	−	−
		F145I	−	−	−	−	−
		I218F	−	−	−	−	−
		Q271E	x	x	x	x	x
		M343I	−	−	−	−	−
		G353V	−	−	−	−	−
		**I356T**^*b*^	x	x	x	x	x
		R371I	x	x	x	x	x
PF3D7_0810800	Hydroxy-methyldihydropterin pyrophosphokinase-dihydropteroate synthase (PPPK-DHPS)	S436A	−	x	x	−	−
		K540E	x	x	x	−	−
		K540N	−	−	−	x	x
		A581G	x	−	−	x	x
PF3D7_1303500	Sodium/hydrogen exchanger	N894K	x	−	−	−	−
		V950G	x	x	x	x	x
		H1375Y	x	−	x	−	−
		H1379Y	−	x	−	x	−
		F1557S	x	x	x	x	x
PF3D7_140800 PF3D7_1408100	Plasmepsin II/plasmepsin III	*amplification*	−	−	−	−	x
PF3D7_1318100	Ferredoxin, putative	**D193Y**^*b*^	x	x	x	x	x
PF3D7_1460900	Apicoplast ribosomal protein S10 precursor, putative (arps10)	**V127M**^*b*^	−	x	x	x	x
		D128H	x	x	x	x	x
PF3D7_1447900	Multidrug resistance protein 2+ (heavy metal transport family) (MDR2)	S208N	x	x	x	x	x
		G299D	x	x	x	x	x
		F423Y	x	x	x	x	x
		**T484I**^*b*^	x	−	x	x	x

a All amino acid changes in italics have recently been associated with ACT treatment failure within the eastern GMS.

b Amino acid changes highlighted with boldface have previously showed strong associations with artemisinin resistance (17).

c The presence (x) or absence (−) of the polymorphism is indicated for each protein.

Increased copy numbers for the multidrug resistance transporter *pfmdr1* and enzymes plasmepsin II/plasmepsin III are known to be associated with enhanced survival of parasites exposed to mefloquine or piperaquine, respectively (29–32). To test if any of the field isolates harbored copy number variations, we created sashimi plots of the next-generation sequencing coverage (Fig. S1 in the supplemental material) and found that plasmepsin II/plasmepsin III are duplicated for isolate APL4G (Table 2). APS3G and APL5G both had copy number variants of *pfmdr1* (Table 2). This suggests that APS3G and APL5G will also likely show resistance to mefloquine, while APL4G might also show resistance to piperaquine (30, 33).

### Variation in PfK13 results in a growth defect in asexual blood stages but not in mosquito stages.

**FIG 1:**
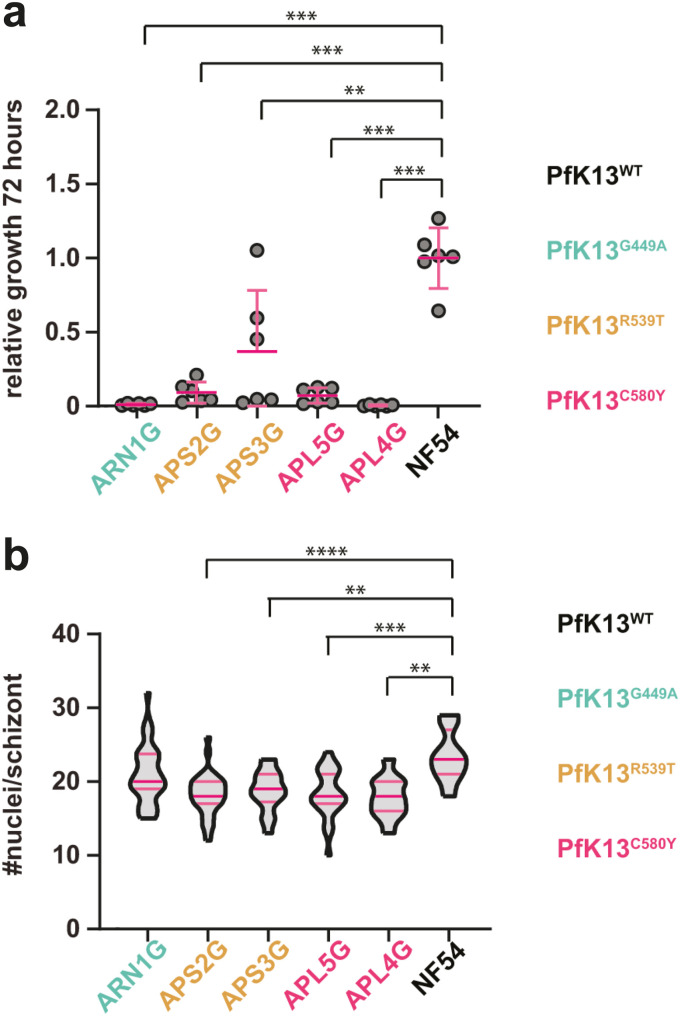
Characterization of P. falciparum clinical isolates. (a) Relative cumulative growth of P. falciparum clinical isolates compared to NF54. Parasitemia was measured by flow cytometry every other day for eight consecutive days (four replication cycles). Six biological replicates from two parallel experiments are shown. Clinical isolates with K13^var^ grow significantly slower than NF54 (K13^WT^, unpaired *t* test, **, *P* < 0.01; ***, *P* < 0.0001). Error bars denote standard deviation (SD). (b) K13^var^ parasites have less nuclei per schizont than NF54 parasites with the exception of isolate ARN1G (unpaired *t* test, **, *P* < 0.01; ***, *P* < 0.0001). Dark pink bars indicate the median and light pink bars denote the interquartile range.

PfK13^var^ that are associated with artemisinin resistance are known to also show reduced asexual blood-stage growth (34, 35). To validate this in selected isolates, parasites were set up in synchronized ring-stage cultures, at a starting parasitemia of 2%, and followed over the course of 8 days. Parasitemia was analyzed every second day by flow cytometry and cultures rediluted to 2%. NF54 parasites showed a cumulative parasitemia as expected under standard laboratory conditions (Figure 1a). Parasites with a PfK13^var^ showed a significantly reduced replication rate in *in vitro* compared to NF54 (Figure 1a), agreeing with previous studies (34, 35). To explore the underlying mechanism of slowed growth, we measured the number of merozoites per schizont, which will directly determine potential growth rates (36). Late synchronized schizonts were blocked from merozoite egress using the protein-kinase G (PKG) inhibitor, compound 2 (37). Thin smears, 12 h later, were then made of each culture and stained with the nuclear stain 4′,6-diamidino-2-phenylindole (DAPI) to count nuclei per schizont. PfK13^var^ isolates displayed fewer nuclei per schizont than the NF54 control, suggesting that the observed reduced growth rate may at least be partially explained by a reduction in the number of progeny (Figure 1b).

To investigate the transmission capability of each P. falciparum field isolate, we induced gametocytes at a starting parasitemia of 2% (38) and, 14 days post induction, fed cultures to Anopheles stephensi mosquitoes by standard membrane feeding assay (SMFA) (39). No significant differences were noted in the stage V (mature) gametocytaemia between different isolates. NF54 showed higher overall stage V gametocytaemia compared to Pf13^var^ (Figure S2a). Male gametocyte exflagellation rates (exflagellation/μl of culture) showed some variability between isolates. Although all the isolates were seeded at equal hematocrit and asexual parasitemia percentages, we cannot directly compare those rates (Figure S2a). Rather, they indicate male gametocyte viability of each individual culture before SMFAs (Figure S2b and c). At 10 days postfeeding, mosquito midguts were dissected and oocyst numbers recorded. All field isolates were found to be capable of infecting mosquito midguts at varied intensity levels, i.e., oocyst counts per midgut, as reported previously for Cambodian field isolates (40). To test if PfK13^var^ led to a reduced replication rate in mosquito stage growth (following the reduced merozoite count), we measured the diameter of each oocyst in these infections as a proxy for replication. Oocyst size showed no consistent pattern of variation compared to NF54, other than a PfK13^R539T^ isolate, which displayed significantly larger oocysts (Figure S2c). This shows that while variations in PfK13 may reduce parasite multiplication rate in the asexual blood stage, they do not appear to directly influence transmission and growth in the mosquito stage.

### Exflagellation sensitivity to different antimalarials among isolates with varied PfK13 genotypes.

**FIG 2:**
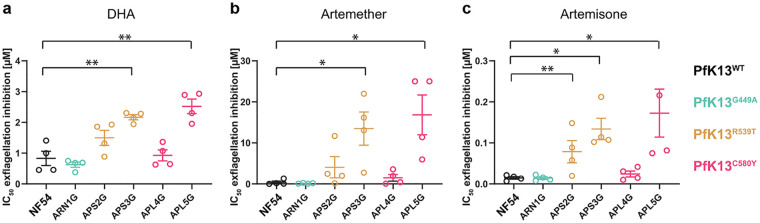
Exflagellation inhibition reported as IC_50_ values of clinical isolates. Two parasite isolates (APS3G and APL5G) show consistent resistance to sterilizing effects of three different artemisinin-related drugs, dihydroartemisinin (DHA) (a) artemether (b) and artemisone (c) on exflagellation compared to the NF54 PfK13^WT^ control. One additional isolate (APLS2G) showed increased resistance to artemisone. IC_50_ values were compared to NF54 (unpaired *t* test, *, *P* < 0.05; **, *P* < 0.01). Open circles denote each biological replicate. Error bars denote the standard error of the mean (SEM).

It has previously been shown that artemisinin and its derivatives have an inhibitory effect on male gametocyte exflagellation, sterilizing male gametocytes from activation, but have no effect on female gametocyte activation (21, 22). To explore whether PfK13^var^ isolates were resistant to this sterilizing effect, we applied a modified dual gamete formation assay (PfDGFA) (22) measuring only the male gamete activation, i.e., male gamete formation assay (MGFA) for this purpose. A 24-h incubation of cultures with the artemisinin derivative DHA was found to be insufficient to elicit a complete inhibition of exflagellation for NF54 parasites (Fig. S3), likely as a result of the instability of the drug, which has been shown to have an *in vitro* half-life of 2.3 h in human plasma at pH 7.4 and 37°C (41). To improve activity and allow for a comparative analysis between isolates, gametocytes were exposed to a second compound dose at 24 h after the first, resulting in a double-dose regimen with a readout after 48 h. Double exposure consistently gave complete inhibition of male activation with DHA at the highest concentration tested (Fig. S3). In parallel, two other artemisinin derivatives and four other antimalarial drugs were tested by the MGFA (Fig. S4 and S5). Exflagellation rates for PfK13^var^ isolates varied in the presence of drug, with the two PfK13^R539T^ and PfK13^C580Y^ isolates consistently showing tolerance to artemisinin derivatives (Fig. 2) but not to other compounds, i.e., UCT048 and NITD609. These two pipeline antimalarials are known to target PfPI4K (42) and PfATP4 (43), respectively. The lack of a consistent pattern of reduced sensitivity to artemisinin-based drugs across PfK13^var^ isolates suggests that *K13* polymorphisms alone likely do not completely explain sensitivity of sexual stages to artemisinin treatment. This observation is corroborated by similar findings with piperaquine resistance, which is mostly, but not always, associated with copy number variants in plasmepsin II/plasmepsin III (30), but has now been associated with specific *pfcrt* mutations *in vitro* (27, 28). In addition, the two isolates exhibiting reduced exflagellation sensitivity to artemisinin-based drugs, namely, APS3G and APS5G, are the only two isolates in our study exhibiting amplification of *pfmdr1* (Fig. S1). This is especially interesting as a reduction in copy number variations of *pfmdr1* were previously found to increase DHA sensitivity in asexual parasites (44, 45). However, the presence of even a single isolate with reduced sensitivity to artemisinin-based drugs in both asexual and sexual stages suggests there is the potential that a resistant strain might be favored for transmission to the mosquito.

**FIG 3:**
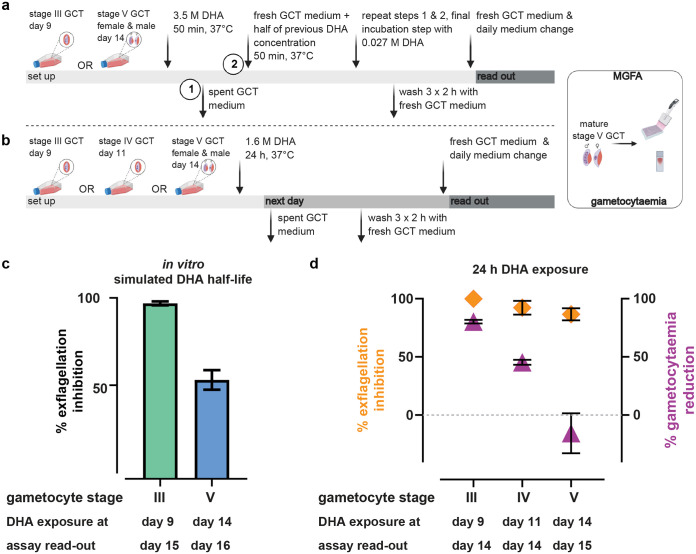
Inhibition of exflagellation is irreversible and independent of a reduction in gametocytaemia for stage V gametocytes. (a and c) Effect of DHA *in vitro* simulated half-life on gametocytaemia and male gametogenesis. (a) Nonpurified stage III (culture day 9) and stage V (culture day 14) gametocytes were exposed to gametocyte culture medium (GCT) containing 3.5 μM DHA or DMSO and culture supernatants were replaced every 50 min with aliquots containing half the DHA concentration of the previous exposure (range 3.5 μM to 0.027 μM). Cultures were then washed stringently and continued until DMSO control cultures reached stage V gametocyte maturity (stage III), or for an additional 48 h (stage V). (c) Exflagellation was assessed and quantified to DMSO controls. (b and d) Irreversible effect of DHA on stage V male gametocytes. (b) Nonpurified stage III, IV, and V (culture days 9, 11, and 14) gametocytes were each exposed to 1.6 μM DHA for 24 h. Cultures were washed vigorously and continued until DMSO control cultures reached stage V gametocyte maturity (stage III and V) or for an additional 24 h (stage V). (d) Exflagellation levels were measured and normalized to DMSO controls (left *y* axis). Gametocytaemia was counted per 1,000 RBC and quantified to DMSO control (right *y* axis). Inhibition of exflagellation was irreversible and independent of a reduction in gametocytaemia for stage V gametocytes. Diamonds and triangles denote the means of biological repeats and error bars denote SEM.

To mimic the impact of DHA’s short elimination half-life (46) on its exflagellation effect, NF54 stage III (early) and stage V (mature) gametocytes were incubated with a single DHA concentration, and the drug replaced with half of the concentration every 50 min (range 3.5 μM to 0.027 μM DHA, a total of eight incubations equaling 6.67 h). A 50-min exposure time was selected as a trade-off between the fast elimination half-life of DHA and the minimum time required for gametocytes to settle by gravity under the assay conditions. DHA was then washed out and gametocytes matured until control cultures reached stage V or an additional 48 h, respectively. When mature stage V gametocytes were exposed to rapidly reduced DHA concentrations, exflagellation was still effectively inhibited (54.72%). Exflagellation of early-stage gametocytes was almost entirely inhibited (Figure 3a and c). This is likely explained through a killing effect, as gametocytes disappear from the culture when early stages are exposed to DHA (Figure 3b and d). Interestingly, the sterilizing effect of DHA on stage V gametocytes appeared to be irreversible, as it was sustained even after the drug had been washed out and was independent from an immediately visible reduction in gametocytaemia (Figure 3d).

### Transmission of field isolates with different PfK13 genotypes under DHA drug selection.

**FIG 4:**
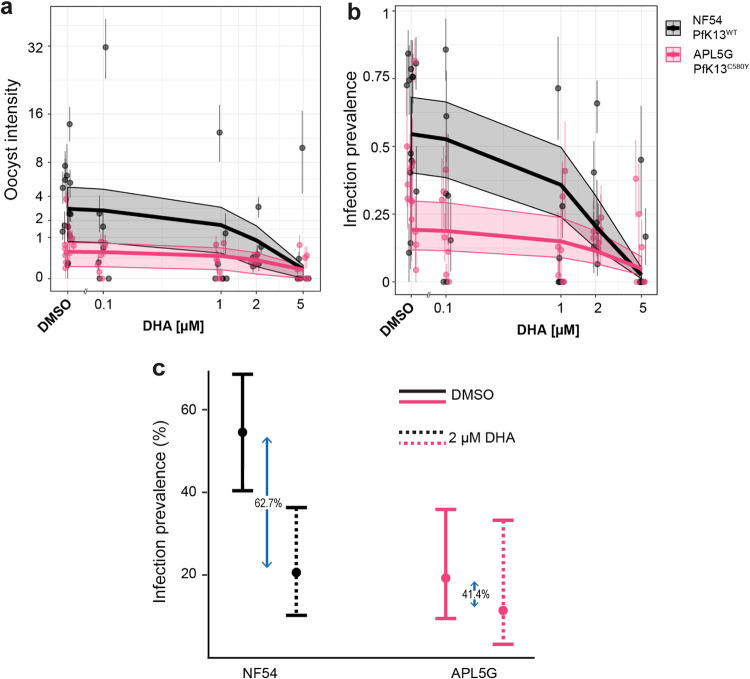
The impact of DHA on the PfK13^WT^ and PfK13^C580Y^ transmission potential. Graphs show the overall results of oocyst counts from 18 individual SMFA experiments measuring oocyst infection intensity (a) and infection prevalence (b) of the sensitive isolate PfK13^WT^ (NF54) versus the artemisinin-resistant PfK13^C580Y^ isolate (APL5G) after incubation with either DMSO (no DHA) or DHA at the specified concentrations. Points and whiskers on each plot show the mean and the bootstrapped 95% CI for each replicate, with the predicted relationship and 95% CI shown with the trend line and shaded region. In the absence of DHA (DMSO), APL5G was predicted to produce significantly fewer oocysts and infections, whereas in the presence of DHA concentrations greater than 2 μM DHA, the transmission potential of PfK13^C580Y^ was comparable to NF54/PfK13^WT^. (c) Fitness costs associated with DHA resistance. The relative reduction in infection prevalence due to DHA treatment in NF54 was greater (62.7%) than in APL5G (41.4%), which suggests that APL5G is significantly more likely to infect mosquitoes under drug treatment (*P* < 0.05) compared to the absence of drug.

To explore the hypothesis that PfK13^var^-associated resistance might allow resistant parasites to efficiently infect mosquitoes under drug coverage, we selected the PfK13^C580Y^ isolate APL5G. This isolate showed a comparable level of mosquito infection to NF54 (Fig. S2) and also showed a high level of male gamete activation resistance to DHA (Fig. 2). Gametocyte cultures of both parasites, APL5G and NF54, were exposed to a range of DHA concentrations for 48 h using our double-dosing regimen, before feeding to A. stephensi mosquitoes by SMFA. The DHA concentration range was selected to ensure exflagellation IC_50s_ for both lines were incorporated in the SMFA three-point dose-response. At day 10 postfeeding, mosquitos were dissected and midguts examined for oocyst load (Fig. S6). Generalized linear mixed effects models were used to analyze infection intensity (number of oocysts per midgut) and infection prevalence (proportion of midguts with oocysts) in response to treatment with DHA, in order to incorporate data from 18 individual SMFA experiments within the same modeling framework. A decrease in both intensity and prevalence of infected mosquitoes was observed for both parasite isolates with increasing DHA concentration (Figure 4a and b). A significant decrease in both the oocyst intensity (ratio of oocyst counts = 0.73, 95% confidence interval [CI]: 0.66 to 0.80) and prevalence (odds ratio = 0.46, 95% CI: 0.41 to 0.52) of mosquito infection was observed for NF54 with increasing drug concentration. In contrast, APL5G parasites (Pf13^C580Y^) showed no evidence for a significant decrease in oocyst intensity with increasing DHA (ratio of oocyst counts = 0.84, 95% CI: 0.65 to 1.07). Increasing concentrations of DHA did still reduce the oocyst prevalence for APL5G (odds ratio = 0.72, 95% CI: 0.56 to 0.96); however, this effect was significantly less than the effect seen for WT parasites (Table 3).

**TABLE 3:** Effects of DHA concentration on oocyst prevalence and intensity^*a*^

Isolate and treatment	Prevalence of infection^*b*^			Oocyst intensity^*b*^		
	Odds ratio	95% CI	*P*	Ratio of oocyst counts	95% CI	*P*
K13^var^ in the absence of DHA	0.20	0.16–0.25	**<0.001**	0.17	0.13–0.23	**<0.001**
DHA (μM): WT line	0.46	0.41–0.52	**<0.001**	0.73	0.66–0.80	**<0.001**
DHA (μM): K13^var^	0.72	0.56–0.96	**<0.001**	0.84	0.65–1.07	0.069

a Analysis was done using generalized linear mixed effects models to incorporate 18 SMFA experiments into a single analysis. Prevalence of infection (presence of at least one oocyst) was modeled using a logistic regression (binomial error structure), with the reference being the artemisinin sensitive NF54 parasites (WT lines) in the absence of DHA. The estimate of 0.46 (0.41 to 0.52) for the impact of DHA on the WT line indicates that for a 1 μM increase in DHA, the odds of a mosquito being infected by a WT parasite are 46%. Oocyst intensity (the number of oocysts) was modeled using a zero-inflated negative binomial distribution. Ratio of oocyst counts refers to the change in the absolute number of oocysts relative to the control, which is the WT line in the absence of DHA. The estimate of 0.17 (0.13 to 0.23) for K13var in the absence of DHA indicates that the expected oocyst numbers for the K13var are 17% compared to the WT.

b CI, confidence interval. Boldface *P* values indicate significance.

To position these findings in the context of likely transmission events, we further explored the impact of DHA on transmission at a single and conservatively selected concentration of DHA (2 μM) (46) in comparison with dimethyl sulfoxide (DMSO). The peak of DHA serum concentration following the recommended WHO ACT oral dosing regimen for artesunate exhibits a wide range with the maximum drug concentration (*C*_max_) up to 7.5 μM (46). Since DHA sterilizes male gametocytes irreversibly, the presence of even a transient spike of 2 μM in serum will, therefore, lead to transmission blocking. In the absence of DHA, NF54 parasites were consistently observed to have a higher infection prevalence (55.1%, 95% CI: 50.5% to 57.8%) compared to APL5G parasites (19.2%, 95% CI: 13.3% to 26.0%) (Figure 4c). This suggests that a fitness cost is associated with the APL5G genotype, which causes a sizeable reduction in the onward probability of infection relative to wild-type (WT) parasites in the absence of DHA in A. stephensi mosquitoes. However, this changed significantly in the presence of DHA. At a concentration of 2 μM DHA, no significant difference was observed between NF54 parasites (20.6%, 95% CI: 27.7% to 14.3%) and APL5G parasites (12.1%, 95% CI: 4.1% to 25.4%) (Figure 4c). The equivalence of infection demonstrates a profound impact of DHA on NF54 but not APL5G transmission, meaning that under clinically relevant artemisinin concentrations, APL5G gametocytes are less affected than NF54 gametocytes for successful onward transmission. This observed transmission-resistance phenotype may offset any fitness costs (such as growth) observed in the absence of drug.

## DISCUSSION

The threat of spreading artemisinin resistance for the treatment of malaria has focused global attention on the mechanisms underlying resistance in the parasite Plasmodium falciparum. However, only limited focus has been placed on how resistant parasites transmit through the *Anopheles* mosquito vector. In this paper, we have shown clear evidence that a clinical isolate with defined artemisinin resistance, based on the known PfKelch13 marker (in terms of clinical delayed clearance and reduced asexual growth sensitivity in the presence of artemisinin-based drugs), is also equally able to transmit to mosquitoes under drug coverage.

In comparison, the wild-type pathogen strain NF54 was shown to be significantly less likely to infect mosquitoes under drug coverage. This suggests that the use of artemisinin will increase the probability of artemisinin resistance being transmitted due to the significant reduction in the probability of wild-type parasites being transmitted. The molecular basis of this transmission-resistance phenotype is likely complex and is clearly not defined simplistically by PfK13 alone. However, the disconnect between PfK13 and transmission resistance is clear from the observation that of the five clinically resistant isolates tested, while each showed clear resistance to artemisinin-based drugs in asexual growth, there was varied sensitivity in transmission stages. However, the transmission-resistance phenotype was nonetheless robust for certain isolates. We initially aimed to compare artemisinin-resistant parasites with locally matched artemisinin-sensitive isolates to allow a more accurate adjustment for the genetic background of the SEA lineages. Unfortunately, at the time of this study, none of the wild-type isolates were producing sufficiently viable male gametocytes and culture adaption of those lines is currently ongoing. Therefore, the canonical NF54 strain, which has been broadly employed in transmission studies, was chosen as the artemisinin-sensitive control.

As with previous studies, we first started with investigation of the asexual growth rate, confirming a consistent reduced rate in resistant isolates. The asexual growth rate reduction seen in PfK13^var^ isolates likely acts as both a selective cost for parasite growth (in being outcompeted in normal infections) but also likely explains how these parasites persist during drug treatment, i.e., explaining delayed clearance (14, 15). Switching our focus to sexual commitment and development, we next explored gametocyte production. With the caveat that different parasite isolates always show marked differences in gametocyte formation capacity, we did not observe any obvious reduced capacity among PfK13^var^ isolates in gametocyte production. All five field isolates produced comparable numbers of mature gametocytes (stage V gametocytes) after day 14 upon induction. Indeed, a positive correlation between drug resistance and sexual commitment has been consistently reported. Patients with delayed parasite clearance due to artemisinin resistance display higher gametocytaemia levels, suggesting an elevated potential for transmission of these parasite isolates (16). While commitment of gametocytes to either male or female is poorly understood, it is entirely conceivable that gametocytes mature or differentiate into either male or female at differing rates in each isolate. Unfortunately, sex ratios were untested here due to a paucity of markers for male and female gametocytes and challenges with definitive differentiation of the sexes using Giemsa stain. Irrespective of this, gametocyte conversion rates have been shown to be sensitive to asexual stage replication, which itself is affected by drugs. This suggests there is the potential for a trade-off between asexual stages and sexual stages in ensuring the spread of the artemisinin-resistant parasites (47).

With sexual commitment and exflagellation *in vitro* seemingly uncompromised in resistant isolates, we next sought to explore transmissibility directly. While we saw differences in infection intensities between the different isolates, the transmission replication rate (as measured by oocyst size) among the five parasite field isolates and NF54 was similar. The latter point is noteworthy, since it is clear that artemisinin-resistant parasite isolates show a lower asexual growth rate and merozoite (progeny) rate (Fig. 1), however, sporogony in the mosquito does not appear to be affected. Thus, PfK13^var^ parasites appear able to commit to sexual reproduction, activate, and transmit to mosquitoes at similar levels as NF54 (i.e., beyond variability usually seen between isolates). Still, it is important to note that further studies are needed to fully address the influence of *K13* genotype on oocyst growth and, ultimately, number of viable and infectious sporozoites.

Shifting our attention to transmission under drug coverage, tests of the viability of gametocytes for gamete activation using the MGFA with artemisinin derivatives; DHA, artemether, and artemisone clearly found that certain PfK13^C580Y^ and PfK13^R539T^ parasites demonstrated significantly higher resistance compared to NF54. Of note, while undertaking this work, a parallel study made similar observations. Testing male exflagellation sensitivity to DHA in unrelated culture-adapted PfK13^var^ Cambodian field isolates, Lozano et al. found that PfK13^var^ isolates showed a reduced sensitivity of exflagellation rates to DHA treatment, though onward mosquito infectivity was not tested (48). Extending this observation to transmission directly, we took the most competent transmissible field isolate representing the resistant phenotype (APL5G, PfK13^C580Y^) compared to the laboratory reference strain NF54, and tested whether transmission resistance plays out in terms of capacity to infect mosquitoes over a range of drug concentrations. Controlling for number of RBCs and hematocrit level for each, infection prevalence in mosquitoes could then be tested and compared between drug treatment of the same parasite isolate. Of note, the oocyst intensity (number of oocysts found in each mosquito) was consistently different between NF54 and APL5G, as it is for each different culture-adapted parasite strain (40). These differences make direct measures of mixed infections challenging. Nonetheless, we found that under DHA pressure, transmission of the artemisinin resistant, PfK13^C580Y^ (APL5G) was consistently less impaired than transmission of NF54 (Figure 4c). This is due to the greater impact of DHA on NF54 oocyst infection, which served to offset the decreased transmission potential for APL5G in the absence of DHA. Our findings demonstrate that the artemisinin-resistant phenotype of APL5G is not confined to asexual blood stages but, additionally, expands to male sexual stages and directly influences transmission in the presence of DHA. APL5G additionally carries a *pfmdr1* amplification which has been previously linked to asexual artemisinin resistance (44, 45). Yet, in a clinical setting, the most prominent multidrug-resistant P. falciparum lineages show very low *pfmdr1* amplification prevalence across the GMS (10, 49). Despite *pfmdr1* copy number variations potentially having an association with increased DHA resistance during gamete formation of APL5G, clinical evidence suggests this amplification is unlikely to have provided a true advantage for wider transmission and manifestation outside the use of the artemisinin partner drug mefloquine (10, 49).

Although the numbers are small here, the implications are that in the context of a mixed infection, a resistant parasite isolate may be more likely to survive ACT treatment and, additionally, its gametocytes may be more likely to transmit to the mosquito. Thus, ACT coverage in the field may be favoring, even driving, artemisinin-resistant parasite persistence and transmission. This could explain an important part of the selection of PfK13 propeller mutants observed in the field. For instance, the F446I PfK13 mutation results in only a slight prolongation in parasite clearance half-life and is not associated with ACT treatment failure (16). Yet, there is clear selection of this genotype in Myanmar and Southern China (50), which could be explained by preferential transmission under artemisinin drug pressure. The effect on outcrossing is also worth considering. Because the sterilizing effects of artemisinin-based drugs only affect the male gametocyte (21, 22), there is the very real potential that ACT usage in the context of a mixed infection might favor acquisition of other selectively advantageous mutations during transmission. Since the female gametocytes remain unaffected, successful transmission under ACT coverage would likely favor either resistant parasite selfing or mating between resistant males and sensitive females. It is clear from our own usage of a central Asian mosquito vector (A. stephensi) and the work of others using the major African vector, A. coluzzii (40), that artemisinin-resistant parasites can infect nonnative mosquitoes. Thus, in a mixed infection where local parasites show a degree of geographical vector adaptation (51), an invasive resistant parasite, otherwise at a disadvantage (reduced vector adaptation and slower asexual growth), may acquire a key advantage under ACT coverage in terms of its ability to both transmit and acquire necessary adaptive mutations via recombination with sensitive females. Importantly, this may play out even without a decline in cure rates if transmissibility of the treated infection is increased, such as in high-intensity transmission areas at the early stages of resistance invasion before partner drug resistance has emerged. Mixed infection studies *in vivo* and modeling of drug coverage effects with different rates of transmission intensity are clearly needed to explore the implications of transmission resistance in various invasive settings.

Ultimately, these data stress the importance of considering transmission in the context of drug resistance spread and argue strongly for the inclusion of a parasite transmission-blocking component in future antimalarial combination therapies or control strategies.

## MATERIALS AND METHODS

### P. falciparum asexual blood stage and gametocyte maintenance.

Asexual blood stage and gametocytes were cultured as previously described (38) with the following modifications. Asexual blood-stage cultures were maintained in asexual culture medium (RPMI 1640 with 25 mM HEPES [Life Technologies], 50 μg liter^−1^ hypoxanthine [Sigma], 5% A+ human serum [Interstate Blood-Bank], and 0.5% AlbuMAX II [Life Technologies]). Gametocyte cultures were maintained in gametocyte culture medium (RPMI 1640 with 25 mM HEPES [Life Technologies], 50 μg liter^−1^ hypoxanthine [Sigma], 2 g liter^−1^ sodium bicarbonate [Sigma], 5% A+ human serum [Interstate Blood-Bank], and 0.5% AlbuMAX II [Life Technologies]).

### Field isolates.

All parasite isolates were sequenced and assessed for multiplicity of infection at point-of-care baseline before ACT treatment. Selected isolates were sufficiently homozygous (based on the value F_WS_ delineated in reference 23), which allowed us to proceed without additional parasite cloning steps. Interestingly, all copy number variations for APS3G, APL5G (mdr1), and APL4G (plasmepsin II/III) were maintained after prolonged culture cultivation and several cryopreservation steps, as the same copy number variations were found at baseline (52) and during our most recent sequence analysis in 2019.

### Mosquito rearing.

Anopheles stephensi mosquitoes were reared under standard conditions (26°C to 28°C, 65% to 80% relative humidity, 12 h:12 h light/darkness photoperiod). Adults were maintained on 10% fructose.

### Whole-genome sequencing.

Genomic DNA isolation, whole-genome sequencing, and calling of single-nucleotide variants were undertaken essentially as recently described (52). To determine gene amplification copy number variants, sashimi plots were created and visualized using the integrated genomics viewer (IGV) (53) comparing aligned bam files. Sashimi plots were visually inspected for increased read coverage over genes of interest.

### Flow cytometry.

To prepare parasites for the growth assay, asexual parasites were sorbitol-synchronized at least twice 16 h apart to create an 8 h growth window. Briefly, cultures containing mainly ring-stage parasites were incubated with 5% sorbitol at 37°C for 5 min, spun down, and resuspended in culture medium. The second synchronization step was repeated 16 h later, resulting in a culture where parasites were between 16 h to 24 h ring stages. To start the growth assay, parasitemia was seeded in 2 ml total volume at 2% hematocrit and 1 to 2% early ring stages in triplicates that were treated separately. The assay was performed twice with 3 replicates each. Every 48 h and for a total of 8 days, parasitemia was determined using flow cytometry and each well was diluted back to 1 to 2% parasitemia as follows. One milliliter of each culture was fixed in 4% formaldehyde and 0.2% glutaraldehyde for at least 10 min. After washing with phosphate-buffered saline (PBS), DNA was stained with SYBR green I (diluted 1:10,000) in the dark for 20 min at room temperature. After incubation, cells were washed three times with PBS and resuspended in 80 μl PBS. Flow cytometry was performed counting a total of 100,000 cells per well. Cumulative growth was calculated based on absolute parasitemia and dilution factor for each day.

### Nuclei count.

Parasites were synchronized twice using 5% sorbitol to obtain a 10-h life cycle window. An aliquot of 10 μM compound 2 was added to late trophozoite stages for a maximum of 12 h to block egress of the RBCs (37). Resulting segmented schizonts were thinly smeared, then fixed with 4% formaldehyde and 0.2% glutaraldehyde for 20 min. Smears were then stained with 1 μg ml^−1^ DAPI for 5 min and mounted in Vectashield (Vector Laboratories). Z-stacks were taken using a Leica microscope at 100× magnification. Nuclei of arrested segmented schizonts were counted using the plugin tool “Manual counting” on ICY (54). Only singly invaded RBC were counted.

### Trophozoite maturation inhibition assay.

The trophozoite maturation inhibition assay (TMI) was performed as described (24). Briefly, P. falciparum-infected blood was collected into heparin-coated vacutainer tubes and centrifuged at 800 × *g* at 4°C for 5 min to allow the removal of the plasma and buffy coat. This was followed by three washes in RPMI 1640 (without serum supplement) and adjusted to 3% cell suspension in 10% A+ human serum-supplemented RPMI 1640. Then, 96-well microtiter plates (Nunc MicroWell 96-well microplate; Thermo Fisher Scientific) were predosed with artesunate dissolved in 5% NaHCO_3_ (Guilin Pharmaceutical Co., Ltd., China), ranging from 0.01 to 400 ng ml^−1^ final concentration or no drug as negative control. A 75-μl P. falciparum ring stage infected RBC cell suspension was added to the test plate and incubated for 24 h at 37°C in 5% CO_2_. All samples were tested in triplicate. Upon completion of drug exposure, thick and thin blood smears were prepared of all wells and the number of 24- to 30-h trophozoites (55) was counted per 100 infected RBCs. To identify the inhibition activity of artesunate, the percentage of trophozoite maturation compared to the negative control was assessed. The IC_50_ (50% inhibitory concentration) was calculated as the drug concentration causing 50% inhibition of P. falciparum maturation from ring stage to trophozoite stage and normalized to the negative-control wells. All IC_50s_ were determined by sigmoid curve fitting using WinNonlin computer software (version 3.1; Pharsight Corporation, USA). As a technical control, all *ex vivo* assays were performed in parallel to the standard laboratory Thai strain TM267, which is a non-gametocyte-producing line.

### Male gamete formation assay double-dose format.

The double dose male gamete formation assay (MGFA) was adapted from previously described methods (22) to incorporate an additional drug dosage, accounting for the low compound half-lives of artemisinin and its derivatives. Briefly, compounds were prepared in 10 mM DMSO stocks and dispensed in serial dilutions into multiwell plates using an HP D300 digital dispenser. All drugs were supplied by the Medicines for Malaria Venture (MMV), including UCT048 (MMV048), NITD609 (Cipargamin), gentian violet, methylene blue, and DHA. Samples were normalized to 0.25% DMSO and contained 0.25% DMSO and 12.5 μM gentian violet as negative and positive controls, respectively. Half the maximal DMSO content was plated per plate, accounting for the accumulation of DMSO over two dosages. Mature gametocytes with an exflagellation rate of >0.2% of total cells were diluted in gametocyte culture medium to 25 million RBCs ml^−1^. Mature gametocyte culture was plated in drugged 96-well plates and incubated in a humidified chamber under 92% N_2_/5% CO_2_/3% O_2_ (BOC special gases) at 37°C for 24 h. For the second drug dosage at 24 h, the drugged culture was transferred to a second drugged well plate and incubated for a further 24 h under 92% N_2_/5% CO_2_/3% O_2_ at 37°C in a humidified chamber.

At 48 h, gametogenesis was induced with ookinete medium (RPMI 1640 with 25 mM HEPES [Life Technologies], 50 μg liter^−1^ hypoxanthine [Sigma], 2 g liter^−1^ sodium bicarbonate [Sigma], and 100 μM xanthurenic acid). Plates were immediately incubated at 4°C for 4 min and then 28°C for 5 min before transferring to a Nikon Ti-E widefield microscope. Exflagellation events were recorded by automated phase-contrast microscopy, in 96-well plates. Twenty-frame time lapses were recorded at 10× magnification and 1.5× zoom. Exflagellation events per field were derived using an automated ICY Bioimage Analysis algorithm. Resulting counts were converted to percentage inhibition values, calculated relative to positive (C1) and negative (C2) controls:

(1) $$\%\mathrm{Inhibition}=100-\left(\left(\frac{\mathrm{test}\;\mathrm{compound}-C1}{C2-C1}\right)\times 100\right)$$

Raw data demonstrated a Z-score ≥ 0.4 and was derived from *n* ≥ 2 and *n* ≥ 3 technical and biological replicates, respectively. GraphPad Prism (version 8) was used to calculate IC_50s_ from the dose response data using with the log(inhibitor) versus response–variable slope (four parameters) function. IC_50s_ were derived from curves demonstrating *R*^2^ ≥ 0.95.

### *In vitro* simulated DHA half-life and wash-out assays.

P. falciparum NF54 gametocyte cultures were seeded according to the MGFA protocol on the same day from the same inoculum and maintained in Nunc EasYFlask cell culture flasks (Thermo Fisher Scientific, Nunclon Delta surface treated, 25 cm^2^). DHA was kindly provided by Medicines for Malaria Venture and prepared at a 10 mM stock solution in DMSO and stored at −20°C until further use.

On the day of the half-life assay, nonpurified stage III (culture day 9) and stage V (culture day 14) gametocyte cultures were assessed for their gametocytaemia with Giemsa smears and stage V also checked for male gametocyte exflagellation. Each culture was split into two Nunc EasYFlask cell culture flasks (25 cm^2^), exposed to 92% N_2_/5% CO_2_/3% O_2_, and cells were allowed to settle for 1 h at 37°C. Culture supernatants were removed and replaced with freshly prepared and prewarmed 10 ml gametocyte culture RPMI containing 0.25% DMSO or 3.5 μM DHA (0.25% DMSO final), exposed to 92% N_2_/5% CO_2_/3% O_2_, and cells were allowed to settle at 37°C. After 50 min, culture supernatants were aspirated and replaced with 10 ml of freshly prepared and prewarmed gametocyte culture RPMI containing DMSO in the control culture or 1.75 μM DHA for the treatment culture (half of the initial DHA concentration). This step was repeated until a final exposure of 0.027 μM DHA was reached (8 exposure steps). Culture supernatants were then replaced with 10 ml of prewarmed gametocyte culture RPMI and incubated for 2 h before replacement of supernatants with fresh gametocyte culture RPMI. This washing step was repeated 3 times in total. Gametocyte cultures were then matured until day 15 (stage III) and day 16 (stage V) culture. Exflagellation was assessed according to the MGFA and inhibitions quantified to DMSO controls.

For single-dose DHA wash-out assays, nonpurified stage III, IV, and V (culture days 9, 11, and 14, respectively) gametocytes were each split into two Nunc EasYFlask cell culture flasks (25 cm^2^) and incubated with 10 ml of freshly prepared and prewarmed gametocyte culture RPMI containing either 0.25% DMSO or 1.6 μM DHA, exposed to 92% N_2_/5% CO_2_/3% O_2_, and kept at 37°C for 24 h. Supernatants were aspirated and all cultures were washed 3 times for 2 h each time, according to the half-life washing steps above. Gametocytes were then further cultured until day 14 (stage III and stage IV) and day 15 (stage V). Exflagellation levels were measured according to the MGFA. Gametocytaemia was counted per 1,000 RBC.

### Standard membrane feeding assay.

Gametocytes were induced and maintained as described above. At day 14 postinduction, gametocytes were spun down at 38°C and resuspended in 5 ml of suspended animation buffer (SA) (56). To ensure that a consistent number of RBCs and gametocytaemia were used for drug incubation for each isolate, gametocytes were magnetically activated cell sorting (MACS)-purified and resuspended in gametocyte medium with 25 × 10^6^ fresh RBCs. DHA or DMSO was added to the desired end concentration into a 10-ml gametocyte culture and added again 24 h later (double-dosing within 48 h). After 48 h, the parasite culture was mixed with fresh blood and human serum and fed to adult A. stephensi mosquitoes using a 3D-printed feeder (39).

### Oocyst counts and size.

At day 10 postfeeding, mosquitoes were dissected, midguts were stained in 0.1% mercurochrome, and then inspected using light microscopy with 10× magnification to count oocysts.

To measure oocyst size, midguts of A. stephensi fed on P. falciparum-infected blood were dissected and fixed with 4% formaldehyde, permeabilized with 0.1% Triton X-100 for 1 h, blocked with 3% bovine serum albumin (BSA) for 30 min, and stained with 1 μg ml^−1^ in DAPI for 3 min. Midguts were washed with 1× PBS and mounted in Vectashield. Images were acquired on a Nikon Ti-Eclipse inverted fluorescence microscope. Images of P. falciparum-infected midguts were captured using the DAPI channel and z-stack imaging to obtain greater depth of oocysts. These stacked images were then processed in ND Processing using the Maximum Intensity Projection option, which then created an image with brighter intensity of the oocysts in every midgut. Oocyst detection was automated by using the Automated Spot Detection program based on the intensity of the oocysts compared to midgut cells (NIS-Elements). The size, diameter, and intensity of each selected oocyst were recorded in an MS Excel file for analysis.

### Statistical modeling of oocyst infection intensity and prevalence.

To assess the impact of artemisinin on the ability of each parasite line to form oocysts, we used generalized linear mixed effects models to incorporate data from different experimental replicates within the same modeling framework. These models have previously been used to model transmission-blocking interventions (57). We modeled either infection intensity or prevalence as the response with treatment (DHA concentration) included as a fixed effect and 0 μM DHA represented by control groups treated with DMSO. The parasite line that was treated (PfK13^WT^ or PfK13^C580Y^) was included as a fixed effect to assess the differential impact of artemisinin on transmission success. The impact of treatment between experimental replicates was allowed to vary at random between replicates. A logistic regression (binomial error structure) was used to model the prevalence of mosquito infection, i.e., the presence or absence of oocysts, and a zero-inflated negative binomial distribution was used to model the intensity of infections, i.e., the numbers of mosquito oocysts (58). The 95% confidence interval estimates were generated for the impact of drug concentration by bootstrapping methodology (with 100,000 replicates).

### Data availability.

Raw experimental data are available on request, while genomic data are publicly available at the European Nucleotide Archive (https://www.ebi.ac.uk/ena). Accession numbers, group ERP121586 and individual: ARN1G (ERS3395814); APS2G (ERS3395811); APS3G (ERS3395812); APL4G (ERS3395813); and APL5G (ERS3395810). The NF54 reference genome is available through https://plasmodb.org/plasmo/.

## Supplementary Material

Supplemental file 1

Supplemental file 2

Supplemental file 3
