# Supplementary material for: Transmission of Artemisinin-Resistant Malaria Parasites to Mosquitoes under Antimalarial Drug Pressure
Source: Antimicrob Agents Chemother. 2020 Dec 16;65(1):e00898-20. doi: 10.1128/AAC.00898-20 (PMC7927852; doi:10.1128/AAC.00898-20)
Supplement: Supplemental file 1 [file AAC.00898-20-s0001.pdf]

Supplementary Information for

Transmission of artemisinin-resistant malaria parasites to mosquitoes under antimalarial drug pressure

Kathrin Witmer, Farah A. Dahalan, Michael J Delves, Sabrina Yahiya, Oliver J. Watson, Ursula Straschil, Darunee Chiwcharoen, Boodtee Sornboon, Sasithon Pukrittayakamee, Richard D. Pearson, Virginia M. Howick, Mara K. N. Lawniczak, Nicholas J. White, Arjen M. Dondorp, Lucy C. Okell, Kesinee Chotivanich, Andrea Ruecker and Jake Baum

Correspondence to: Jake Baum (jake.baum@imperial.ac.uk), Department of Life Sciences, Imperial College London, Exhibition Road, South Kensington, London SW7 2AZ, United Kingdom.

This PDF file includes:

Supplementary Figures S1 to S6

Other supplementary materials for this manuscript include the following:

Datasets S1 to S2

Supplementary Figures

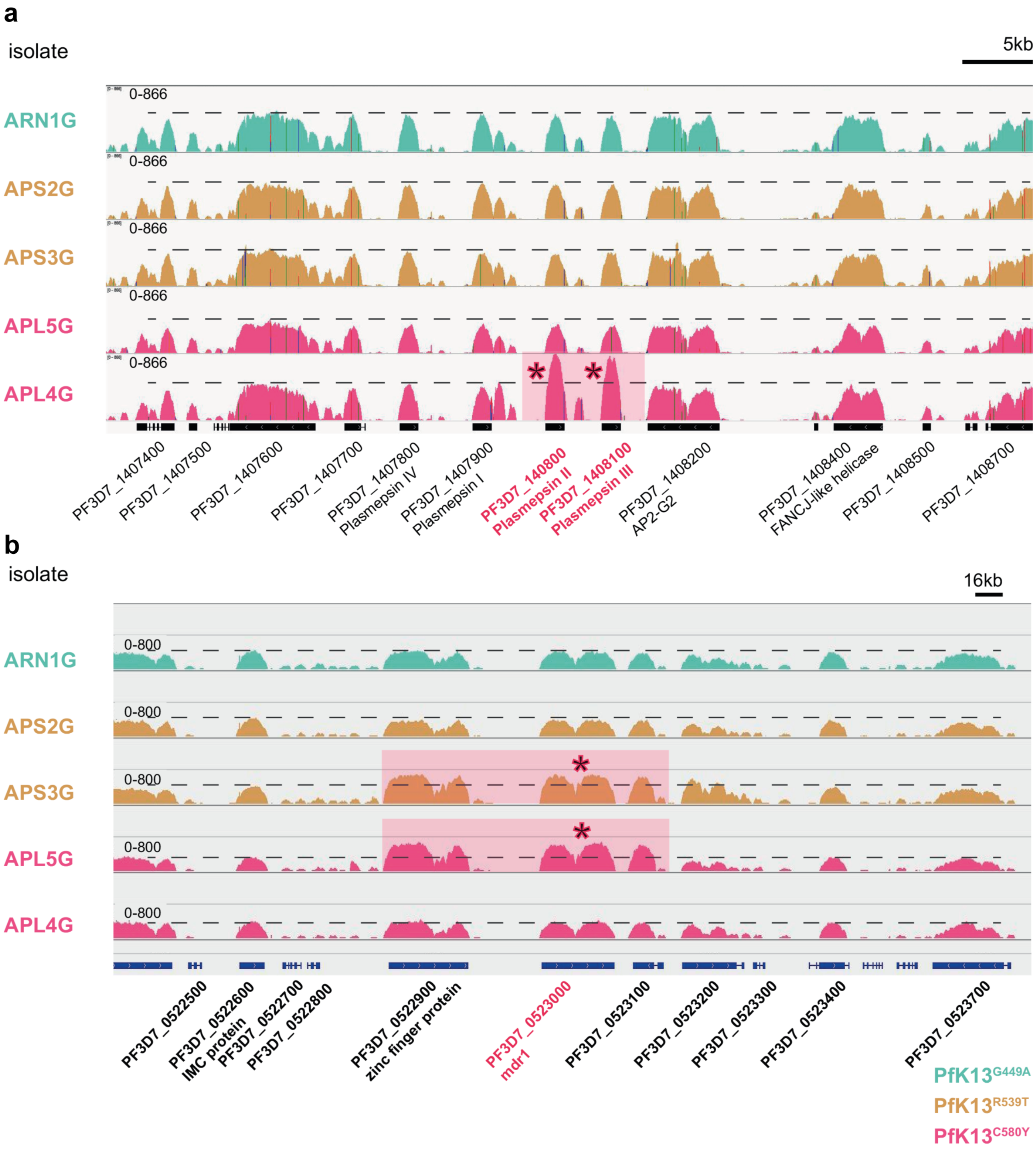

Figure S1. Sashimi plots of field isolates displaying DNA-sequencing coverage over a genomic region of **a.** chromosome 14 and **b.** chromosome 5. **a**. Plasmepsin II/plasmepsin III are highlighted in pink, and asterisks indicate a genome duplication event for these two genes in isolate APL4G. **b**. *Pfmdr1* is highlighted in pink, and asterisks indicate a genome duplication event for the gene in isolates APS3G and APL5G. Dashed line indicates raw DNA-seq pileup reads for the surrounding genes. Parasite isolates and sashimi plots are highlighted according to PfK13 variant.

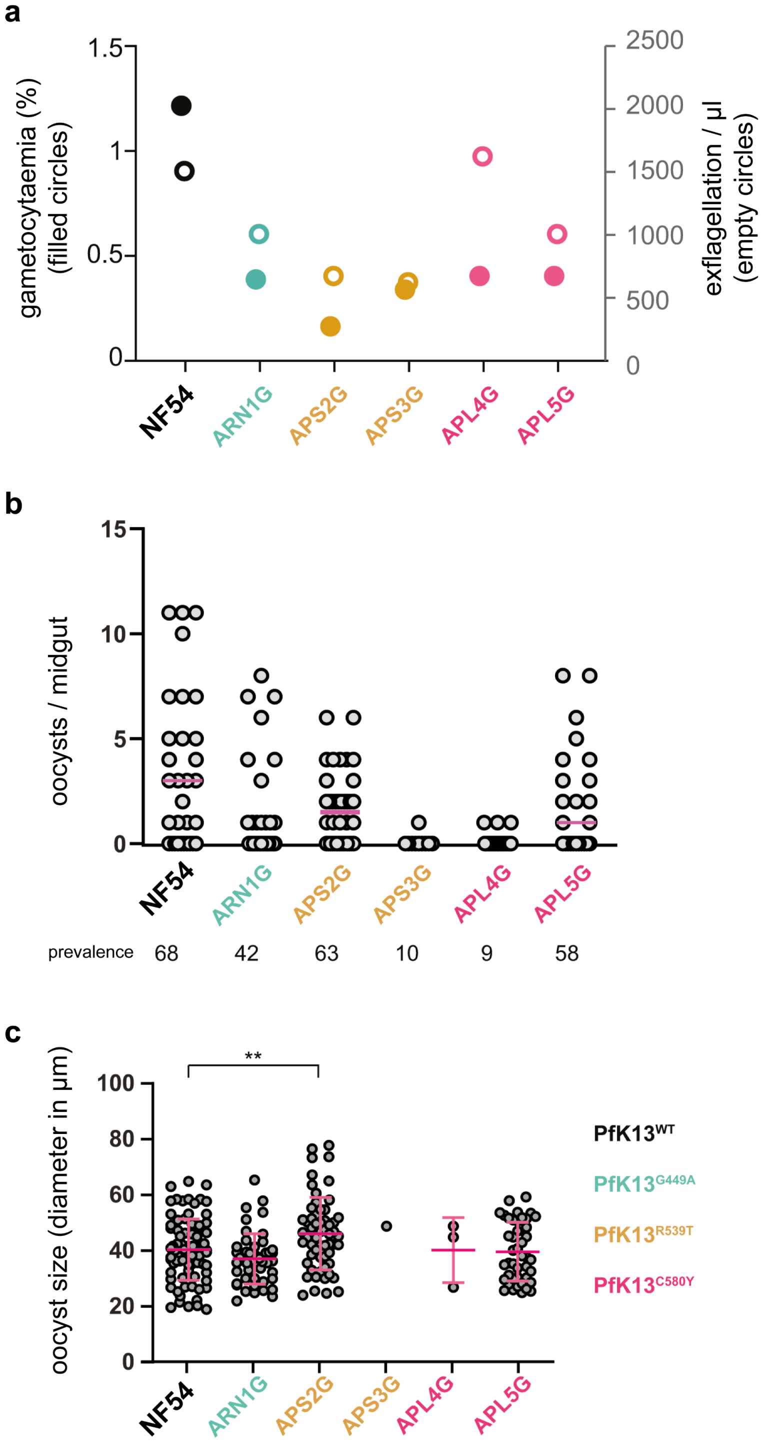

Figure S2. Field isolates form similar-sized oocysts. a. Gametocytaemia (represented by filled circles) and exflagellation rates per µl of culture (represented by empty circles) of five field isolates on day 14 post gametocyte induction. **b.** *An. stephensi* mosquitoes were infected with field isolates, and oocyst numbers and prevalence are shown. **c.** The size of day 10 oocysts was measured in **b**. Error bars denote standard deviation. Graph shows diameter of each oocyst found. PfK13 genotype is not related to oocyst size. Isolate APS2G shows significantly bigger oocysts than NF54 (unpaired t-test, ** p<0.01).

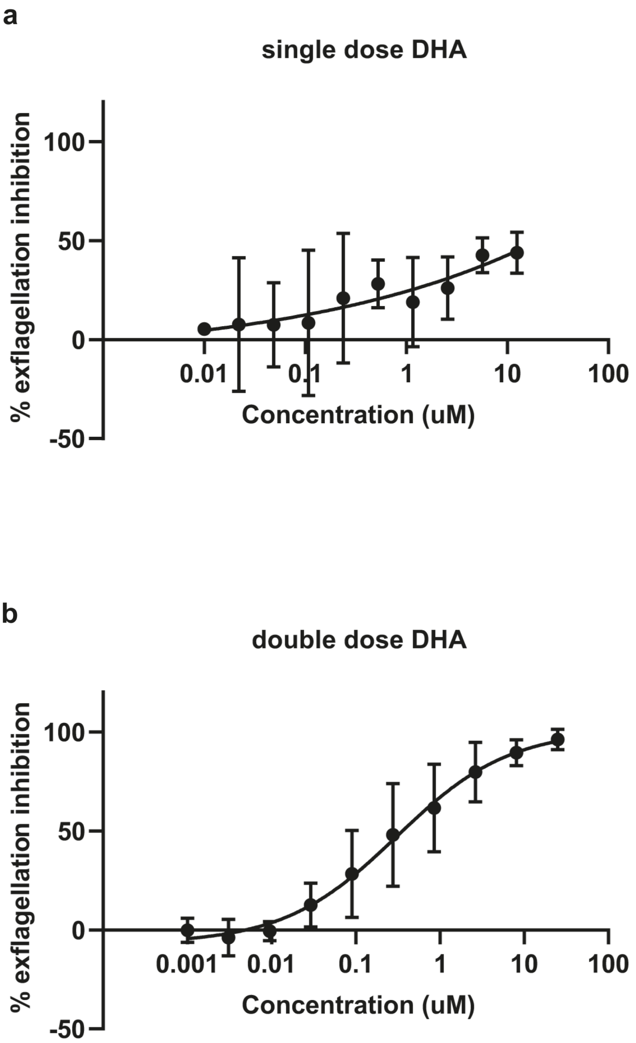

Figure S3. Double-dose DHA elicits a more stable dose-response curve measuring exflagellation inhibition. **a.** NF54 stage V gametocytes were incubated with increasing concentrations of DHA for 24 hours. After incubation, exflagellation centres were counted to give an estimate of % exflagellation inhibition of DHA. Results showed to be very unstable. Three independent biological replicates are shown. **b.** NF54 stage V gametocytes were incubated with increasing concentrations of DHA for 24 hours, after which incubation with the same concentration was repeated for another 24 hours. After 48 hours, exflagellation centres were counted to give an estimate of % exflagellation inhibition of DHA. Results are more stable than with the single dose regimen. Three independent biological replicates are shown. Error bars denote SEM.

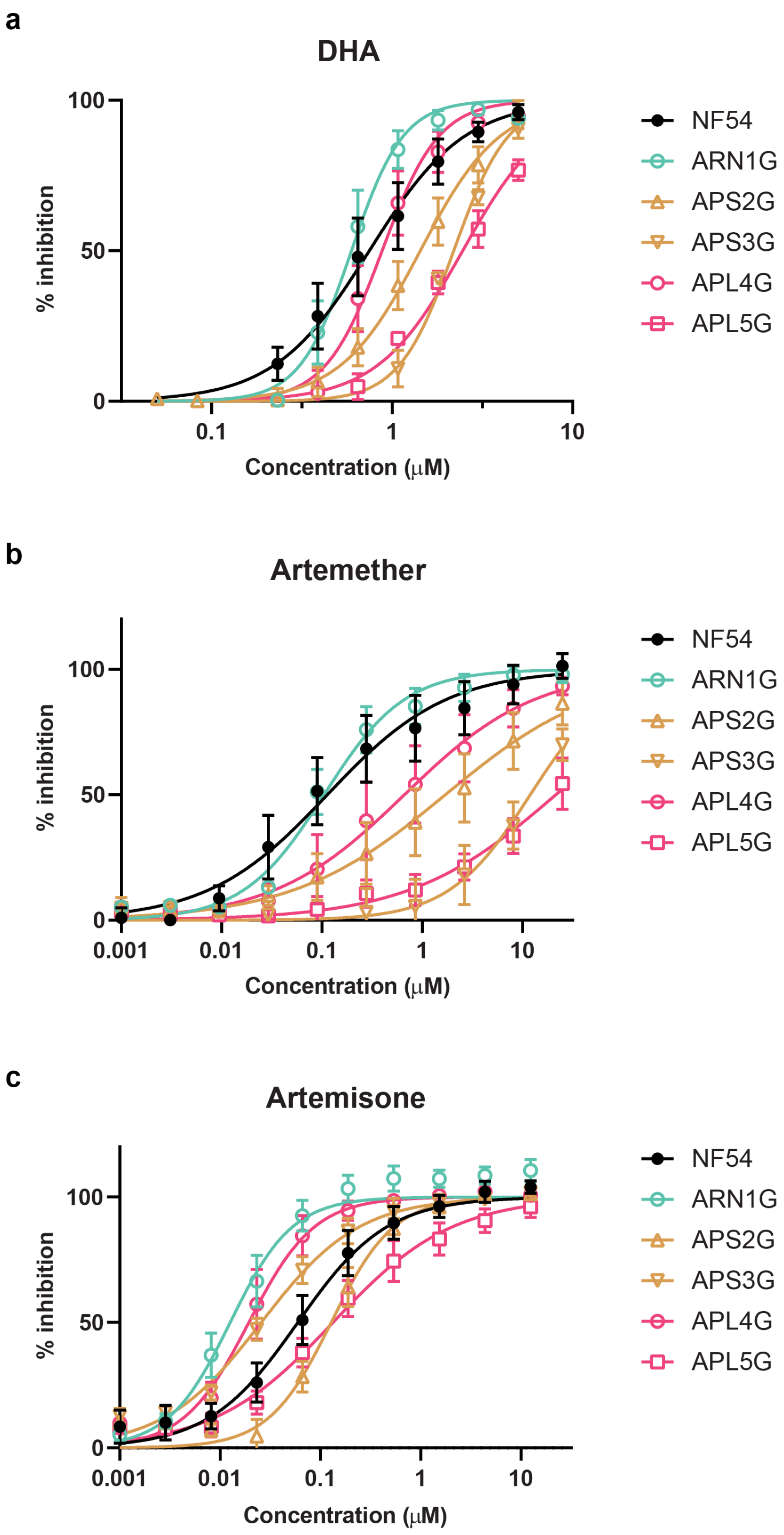

Figure S4. Dose-response curves of three artemisinin-derivatives and their effect on exflagellation inhibition. a. DHA. **b.** artemether. **c.** artemisone. Error bars denote SEM.

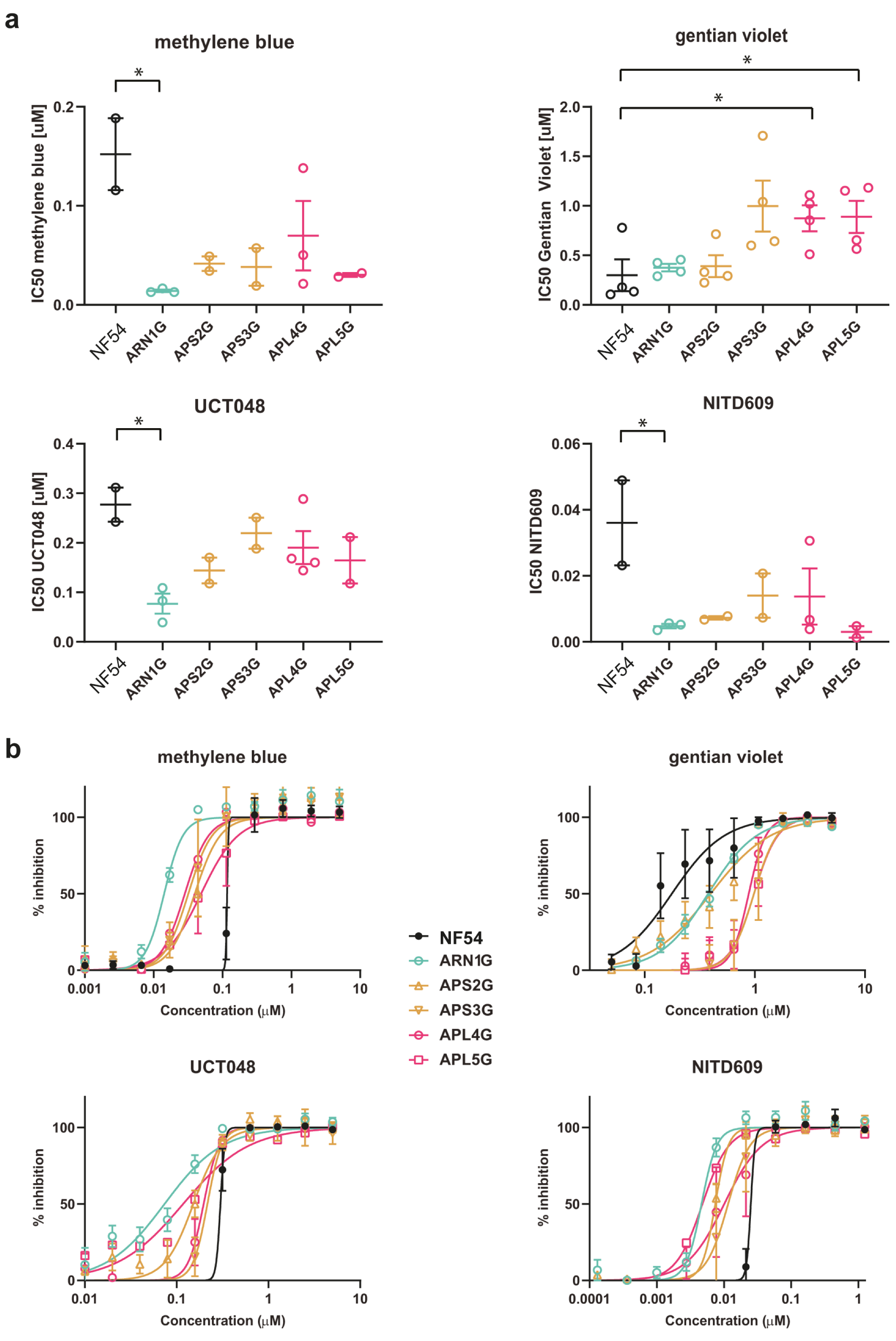

Figure S5. Dose-response curves of four antimalarial compounds and their effect on exflagellation inhibition. a. IC_{50} values of exflagellation inhibition. Open circles denote each biological replicate. **b.** Drug curves. Open symbols denote mean of biological replicates. Errors bars denote SEM.

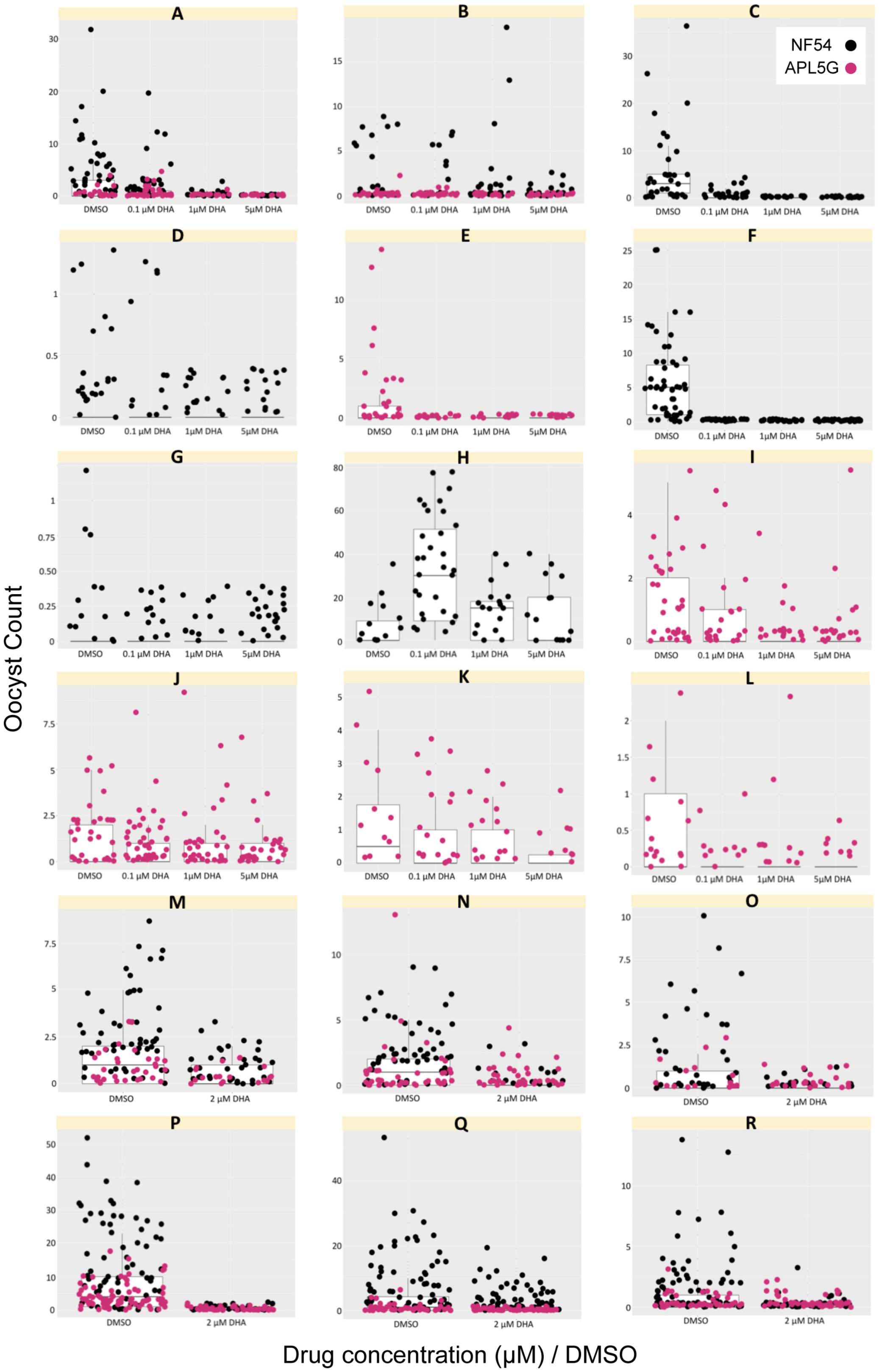

Figure S6. Overall *P. falciparum* infection intensity in 18 individual SMFA (Feed A-R). Each dot represents a single midgut dissected with the number of oocysts plotted on the graph. **A-L.** DMSO, 0.1 µM DHA, 1 µM DHA, and 5 µM DHA were added to **NF54** (black) and **APL5G** (red). **M-R.** Incubation for 48 hours pre-feed with DMSO and 2 µM DHA. Boxplots indicate the median and 25th

-75th percentiles of the oocyst number in every midgut. Due to low oocysts numbers (0 or 1 oocyst per midgut) or because counts were comparatively lower than the control (DMSO), some SMFA data could not be displayed as boxplots.

Supporting Data Sheets (separate files)

Excel spreadsheets of:

1. Dataset S1. MGFA data – male exflagellation data
2. Dataset S2. SMFA data – oocyst counts
